# Supplementary material for: Generation of Endotoxin-Specific Monoclonal Antibodies by Phage and Yeast Display for Capturing Endotoxin
Source: Int J Mol Sci. 2024 Feb 15;25(4):2297. doi: 10.3390/ijms25042297 (PMC10889169; doi:10.3390/ijms25042297)
Supplement: Supplementary file 1 [file ijms-25-02297-s001.zip › ijms-2812205-supplementary.pdf]

Supplementary Information

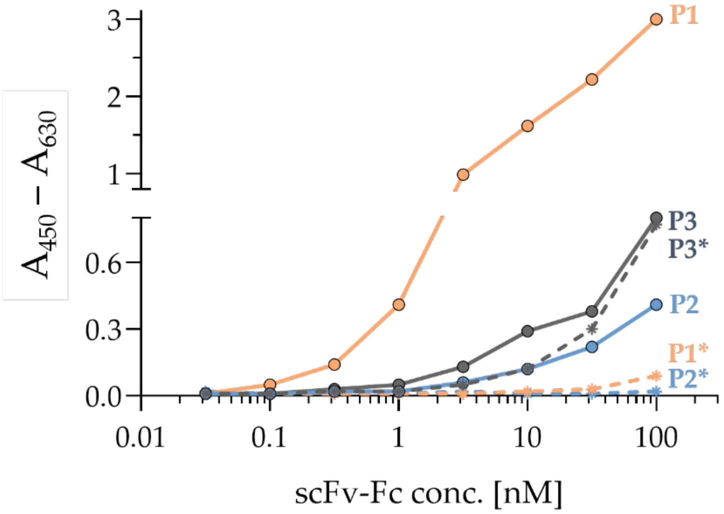

Figure S1: ELISA of different scFv-Fc concentrations (0.03, 0.1, 0.3, 1, 3.2, 32, 100 nM) of clones P1, P2 and P3 either on immobilized biotinylated LPS (solid line) or on streptavidin coated plates (* with dashed line). BSA was used as a blocking reagent. P3* was excluded for further analysis because of low signal to noise ratio.

Table S1: EC_{50} values of Figure 1e that were determined using Graph Pad Prism 8.2.1 analyzing fit agonist vs response (three parameters). Values of aLA are estimated values and have to be treated with caution since saturation of the curve was not achieved.

**Samples**	**P1**	**P2**	**aLA**
EC_{50}	4.4	47.3	267.3

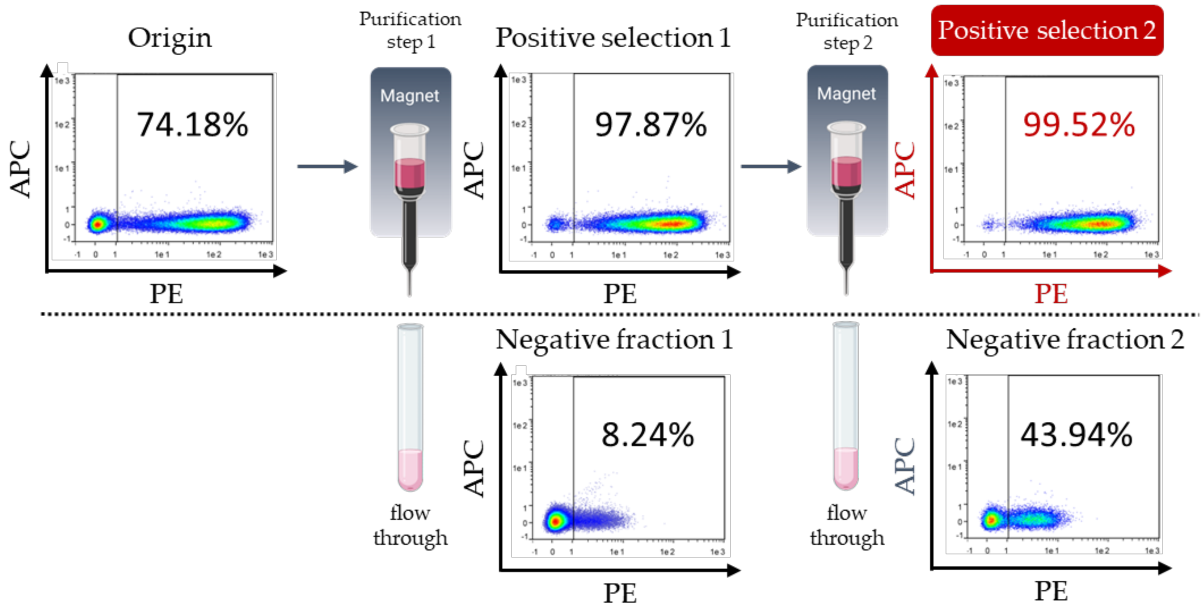

Figure S2: Anti-HA-PE-stained yeast cells were magnetically labeled with anti-PE MicroBeads and positively selected by two subsequent magnetic enrichment steps. Purity was controlled using flow cytometry. Flow-through (negative fraction) showed mainly unlabeled or faint PE-positive cells.

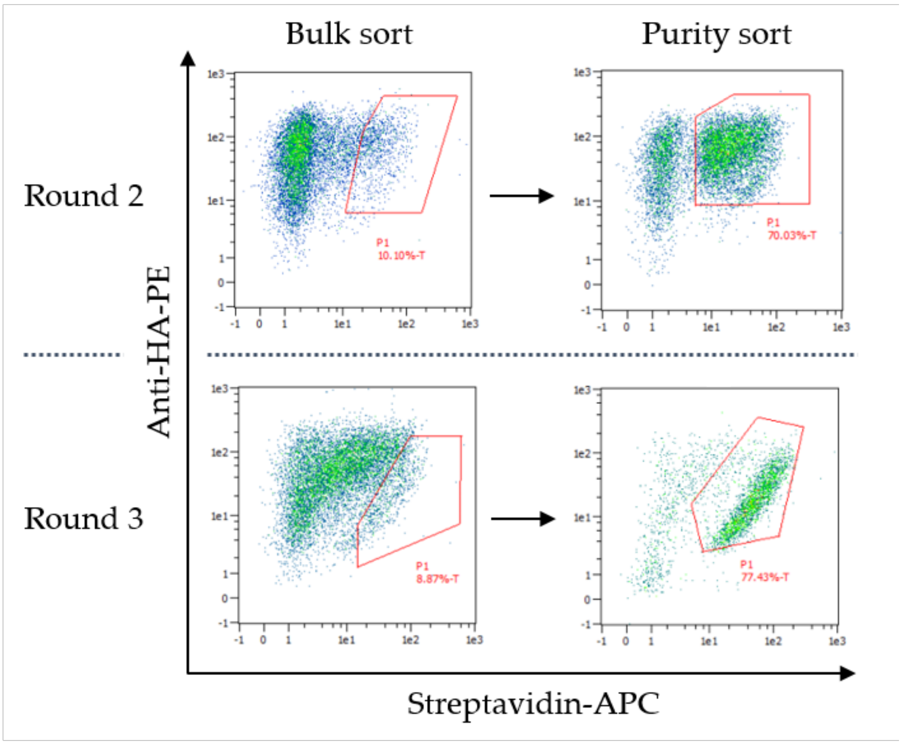

Figure S3: For selection round 2 and 3, LPS-biotin-binding yeast clones were sorted twice on the same day to further increase the specificity of LPS binding yeast cells. Sorting strategy in round 3 was slightly adjusted to sort preferentially on cells with very bright Streptavidin-APC signals resulting in an enrichment of clones with an enhanced likelihood of LPS-binding capacity.

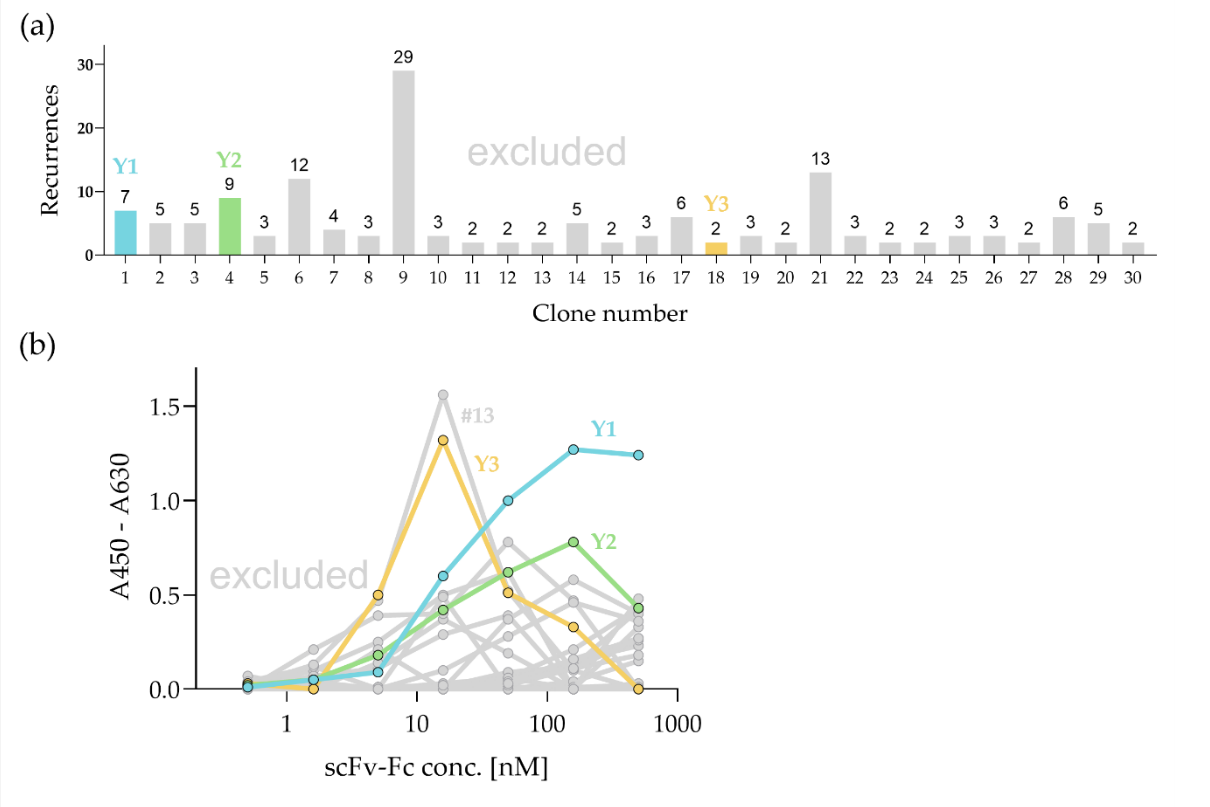

Figure S4: A) 30 out of 128 total clones were found several times. B) ELISA of the 30 scFv-Fc that were found >1 after selection round 3 within 128 randomly picked clones. Absorbance values were calculated by subtracting streptavidin (background) signals from biotinylated LPS signals for each clone. Clones selected for further analysis are shown in color, excluded clones are shown in grey.

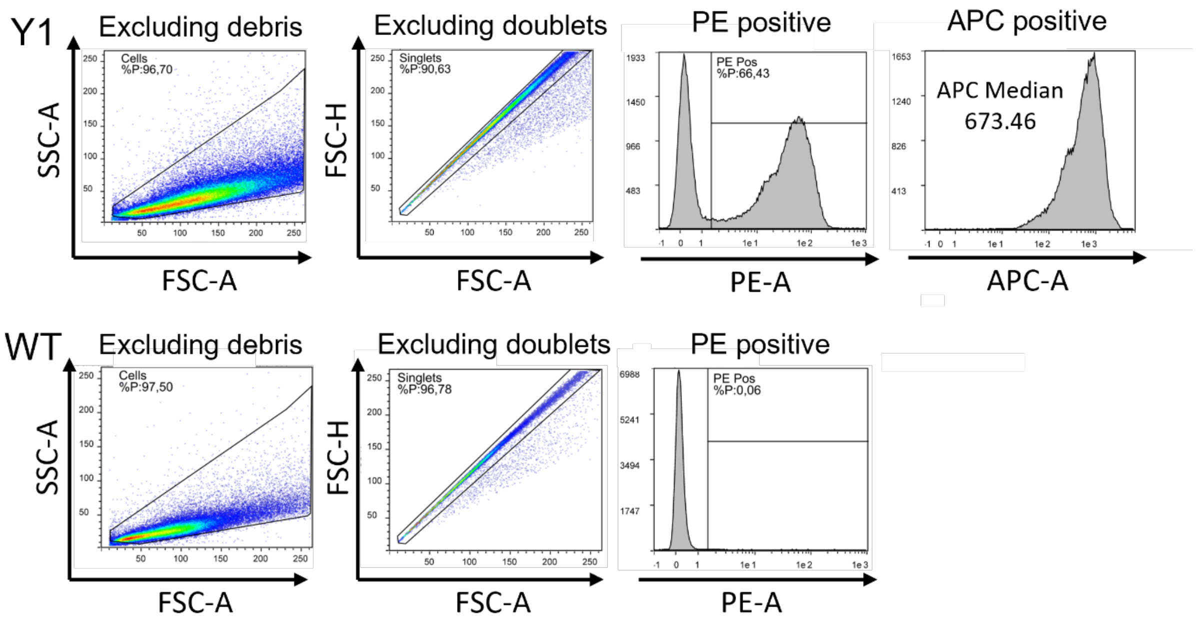

Figure S5: Gating strategy to determine the Median Fluorescence intensity (MFI) of values shown in **Figure 3c**. Y1 is used here as a representative example. After excluding debris and doublets of yeast cells, anti-HA-PE positive cells were gated for positive PE signal while using stained wild type yeast (WT) as a negative control. From PE-A positive cells, the MFI of Streptavidin-APC was determined for each LPS concentration.

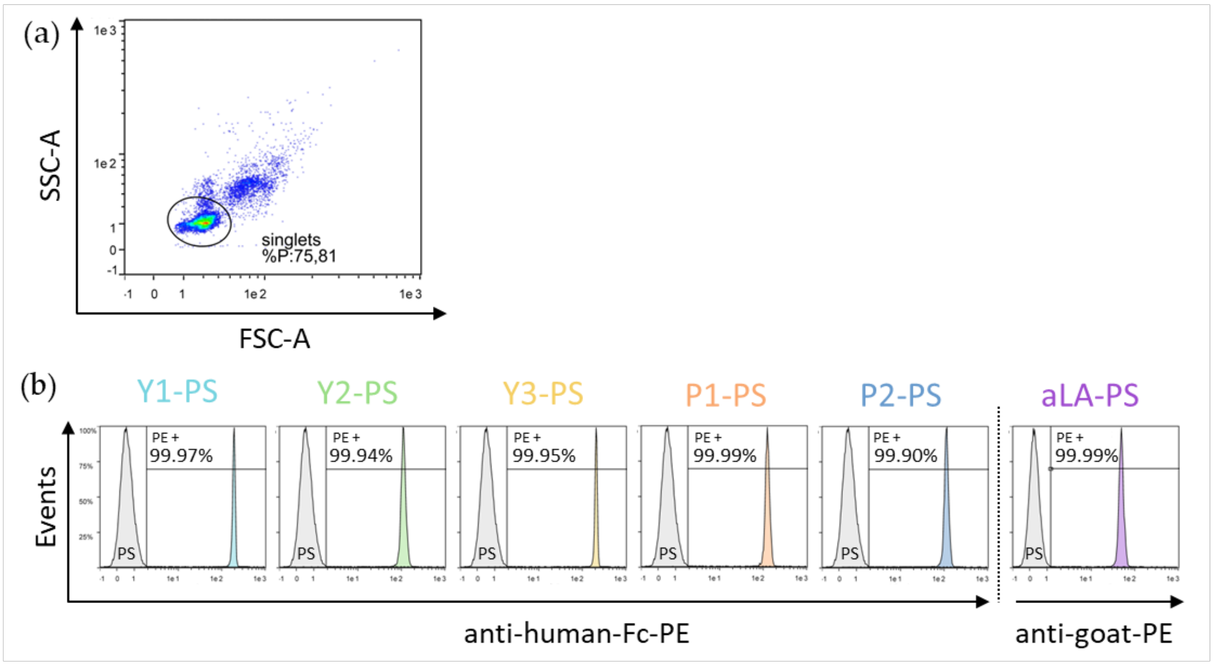

Figure S6: Ab-PS and PS control beads were stained with either anti-human-Fc-PE or anti-goat-PE to measure the conjugation efficiency in flow cytometry; (a) SSC-A vs FSC-A dot plot of Y1-PS as a representative gating for the main population; (b) PS beads (grey) were used to set-up the positive gate (stained with either anti-human-Fc or anti-goat PE, respectively) and merged in an overlay histogram with Ab-beads. All conjugated beads showed staining frequencies equal to or higher than 99.9%.

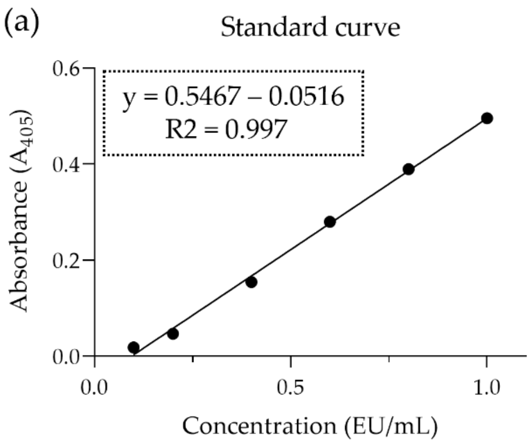

(b)

Sample name	Absorbance A405	Measured conc. (EU/mL)	Dilution	Corrected conc. (EU/mL)
LPS Control 1:60,000	0.457	0.93	60000	55818.5
Y1 No dilution	0.1975	0.46	1	0.5
Y2 1:5,000	0.0375	0.16	5000	814.9

Figure S7: Representative analysis of LAL assay data; (a) LAL calibration curve of standard LPS provided from Pierce Chromogenic Endotoxin Quant Kit (#A39553). A fresh calibration curve was generated for each individual experiment; (b) Processed raw data (absorbance at 405 nm) led to the determination of the remaining LPS concentrations in EU/mL. The measured concentration was adjusted by multiplying it with the dilution factor to obtain the actual data points. Resulting mean data is shown in **Table S2**.

Table S2. LPS in EU/mL after incubating 60,000 EU/mL LPS from *E. coli* O111:B4 with different Ab-beads in water. Values correspond to data shown in Figure 4a. LPS without beads (LPS) and unconjugated beads (PS) were used as controls. Data are mean ± SEM, n ≥ 3.

Samples	LPS	PS	Y1-PS	Y2-PS	Y3-PS	P1-PS	P2-PS	aLA-PS
Remaining LPS (mean) EU/mL	40759.2	27945.4	0.3	253.7	1.5	311.2	1069.5	79.4
SEM EU/mL	6674.4	2999.0	0.08	204.0	0.07	304.4	726.2	60.6

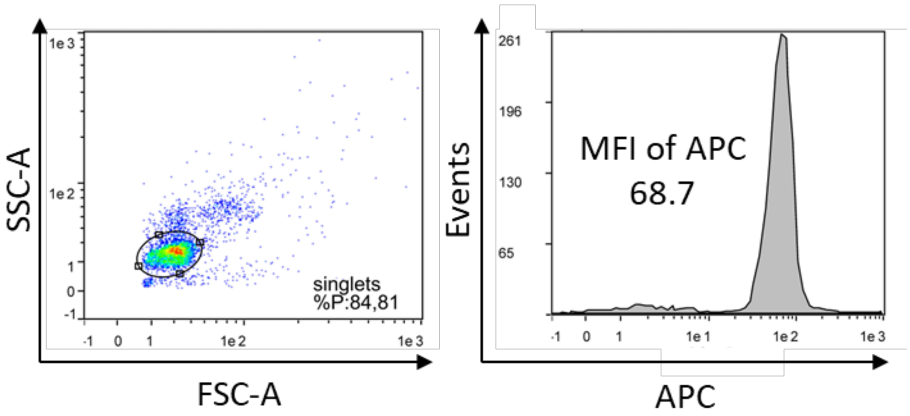

Figure S8: Supplementary plots to Figure 4b. The figure shows Y1-PS as a representative at a concentration of 200 kEU/mL biotinylated LPS. FSC-A vs. SSC-A plots were gated on the main population, and the mean fluorescence intensity (MFI) of Streptavidin-APC was subsequently determined.
